# Assessing the Environmental-Health-Economic Co-Benefits from Solar Electricity and Thermal Heating in Ulaanbaatar, Mongolia

**DOI:** 10.3390/ijerph19116931

**Published:** 2022-06-06

**Authors:** Hooman Farzaneh, Mehrnoosh Dashti, Eric Zusman, So-Young Lee, Damdin Dagvadorj, Zifei Nie

**Affiliations:** 1 Interdisciplinary Graduate School of Engineering Sciences, Kyushu University, Fukuoka 816-8580, Japan; dashti.mehrnoosh.975@m.kyushu-u.ac.jp (M.D.); nie.zifei.940@s.kyushu-u.ac.jp (Z.N.); 2 Transdisciplinary Research and Education Center for Green Technologies, Kyushu University, Fukuoka 816-8580, Japan; 3 Institute for Global Environmental Strategies (IGES), Hayama 240-0115, Japan; zusman@iges.or.jp (E.Z.); lee@iges.or.jp (S.-Y.L.); 4 National Institute for Environmental Studies (NIES), Tsukuba 305-8506, Japan; 5 Climate Change and Development Academy, Ulaanbaatar 14210, Mongolia; damdin.davgadorj@gmail.com

**Keywords:** co-benefits, climate change, solar energy, health impact assessment

## Abstract

This article quantifies the environmental, health, and economic co-benefits from the use of solar electricity and heat generation in the Ger area (a sub-district of traditional residences and private houses) in Ulaanbaatar (UB), Mongolia. The quantification of the featured co-benefits is based on calculating emissions reductions from the installation of the solar photovoltaic (PV) and solar water heaters. A user-friendly spreadsheet tool is developed to shed much-needed light on the steps involved in estimating these co-benefits. The tool simulates the hourly electricity and thermal energy generation, taking into account local meteorological conditions, local geographical data, and technical specifications of the solar power and heat generation systems. The tool is then employed to evaluate two intervention scenarios: (1) Installing 100 MW solar electricity, including both rooftop PV and community grids, to reduce the peak-load burden on the grid; (2) Providing solar thermal heaters for 20,000 households to replace the heating load demand from the existing heat only boilers (HOBs) in UB. The modelling results reveal a significant reduction in GHG emissions and fine particulate matter (PM_2.5_) (PM that is 2.5 microns or less in diameter) by 311,000 tons and 767 tons, respectively, as well as nearly 6500 disability-adjusted life years (DALYs) and an annual saving of USD 7.7 million for the local economy. The article concludes that the mainstreaming spreadsheet-based estimation tools like the one used in this article into decision-making processes can fill important research gaps (e.g., usability of assessment tools) and help translate co-benefits analyses into action in Mongolia and beyond.

## 1. Introduction

### 1.1. Background

**Figure 1:**
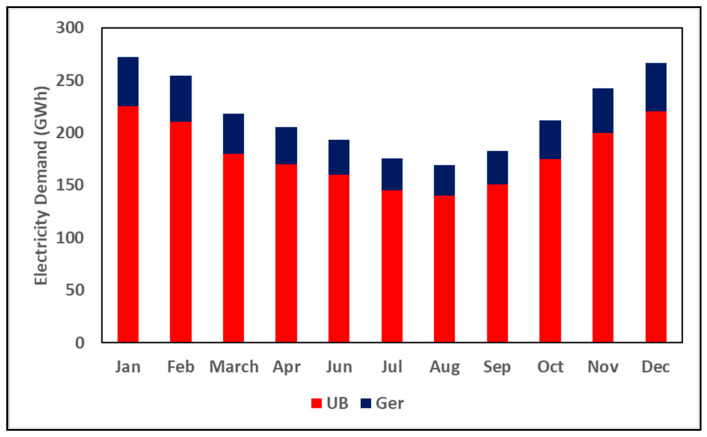
Electricity demand in Ulaanbaatar (Adapted from ref. [5]).

During the winter, Ulaanbaatar (UB), Mongolia, consistently ranks among the cities with the world’s most polluted air [1]. Despite modest improvements in air quality lately, continual inward migration to UB and a subsequent rise in energy use could lead to a resurgence in dangerous pollution levels [2]. UB’s air pollution problems are partially attributable to a heavy reliance on coal, wood, and dangerous non-conventional fuels (including household waste) for heat in UB’s low-income Ger districts (a sub-district of traditional residence houses and private houses) [3]. The Ger residents use various traditional stoves and low-efficiency cast iron HOBs. For example, many HOBs retain heat for only three hours after firing and emit up to 20 times the pollution levels of an efficient stove [4]. Furthermore, UB receives power from five coal-fired power plants with a low thermal efficiency of 35% that can lead to a net increase in both air pollution and CO_2_ emissions. Although most of the houses in Ger districts are connected to the grid, transmission restrictions cause load shedding during peak load periods and temporary blackouts to cope with excess demand. This is especially true during the winter, where the additional load from space heating (4 kW per each household) adds a significant burden to the grid. Figure 1 shows monthly electricity consumption in UB.

**Figure 2:**
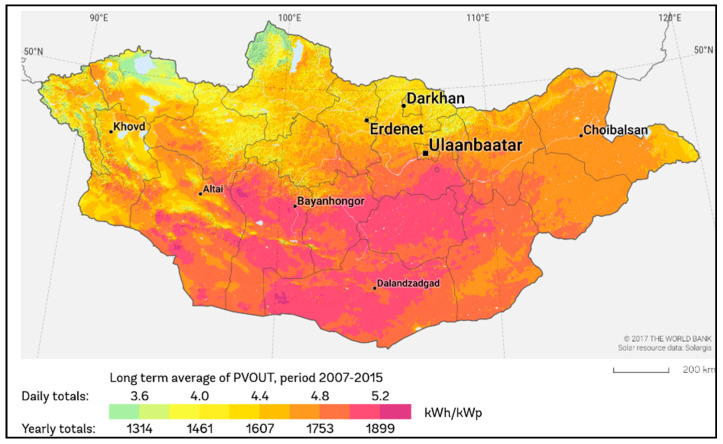
Mongolia potential for solar energy [6].

To achieve both climate mitigation and energy security goals in UB, utilizing more renewable energy sources, especially solar energy, is a pressing need. While coal has dominated Mongolia’s current economy and energy generation, the use of solar energy is rising. This increase is promising because Mongolia has 300 sunny days per year and annual solar radiation of 1700 kW/m^2^ (See Figure 2). Some suggest that UB could generate 150 GW of solar electricity, approximately 15% of the total electricity demand in this city from solar.

Recent policy statements suggest that Mongolia is willing to depend more heavily on solar power. Mongolia joined the Paris Agreement in 2016 and pledged to increase the country’s energy from renewable sources to 20 percent by 2020 and 30 percent by 2030 [7]. To achieve this goal, the government has approved construction licenses for 247 MW of solar energy. In addition, in 2017, the Green Climate Fund (GCF) approved a project to develop a 10 MW solar photovoltaic (PV) farm in the Sumber Soum district of Mongolia [8]. The project is expected to create 15,395 megawatt-hours (MWh) of power per year, reducing 12,270 tons of tCO_2_eq in yearly greenhouse gas (GHG) emissions, while delivering environmental and social co-benefits. The solar farm has increased private sector involvement, making future renewable energy projects easier to finance with domestic private resources [9]. In addition, Sermsang Power Corporation Public Company Limited (SSP) and Tenuun Gerel Construction LLC (TGC) signed an $18.7 million loan with the Asian Development Bank (ADB) and Leading Asia’s Private Infrastructure Fund (LEAP) to develop and operate a solar power plant (15 MW), feeding electricity to Mongolia’s central grid system. The solar power plant, located in Tuv aimag (province) Sergelen soum in the Khushig valley (county), is supposed to provide 22.3 gigawatt-hours annually in Mongolia and lower CO_2_ emissions by 26,400 tons per year. This will help the government in increasing renewable energy’s proportion of total installed capacity from 12% in 2017 to 20% by 2023 and 30% by 2030 [10].

Although Mongolia has pledged to rely more heavily on renewable sources, high-interest rates and unreasonably short tenures have limited renewable energy investment. In addition, though there have been some strides in policy, stronger regulatory signals could attract more investment. However, for many investors and policymakers, the costs of renewable energy are not as great as the benefits. This perceived lack of benefits is partially attributable to the fact that the climate benefits of renewables are long-term, global, and uncertain. They would, therefore, not necessarily accrue to Mongolia. It is also partially attributable to the fact that the health and local economic benefits of renewable energy investment are not explicitly considered in investment and policy decisions. A critical step in accounting for these additional benefits or *“co-benefits”* is quantifying their magnitude.

The interest in quantifying co-benefits started when the term itself was coined in the early-1990s [11]. At that point, co-benefits were often viewed as the additional development benefits of climate actions. This framing was used to convince policymakers in *developed countries* to invest in GHG mitigation since it could improve air quality, health, and address other development needs [12,13]. Co-benefits have since found their way into discussion in *developing countries*—with more emphasis on achieving sustainable development that could also have co-benefits for climate change [14,15,16]. As views on co-benefits evolved, some have suggested the term refers to all *“benefits of policies that are implemented for various reasons at the same time, including climate change mitigation, acknowledging that most policies designed to address GHG mitigation also have other, often at least equally important, rationales”* [17]. 

The consideration of a full range of climate and sustainable development co-benefits continued to gain momentum with the approval of the Agenda 2030 for Sustainable Development (and its 17 Sustainable Development Goals (SDGs)) and the Paris Agreement in 2015 [18]. Both agreements underlined the importance of the integration between sustainable development and climate change. Co-benefits also gained support as high-level reports and well-cited articles demonstrated the usefulness of accounting for co-benefits in a wider range of mix of policies and actions [19,20,21,22,23]. Based on these advances, development banks such as the ADB have begun to assess a suite of co-benefits in carbon finance funds, including new jobs, improved education facilities for children, gender equity, improved energy efficiency, and access to health services. Meanwhile, the GCF (2022) [24] has underlined that recognizing co-benefits is important to ensure that funded activities adhere to sustainable development criteria. 

Cutting across different views and application of co-benefits is an emphasis on estimation and quantification. The steady interest in quantification reflects the long-held belief that what gets measured gets counted in investment and policy decisions. Many studies have since estimated the climate and other co-benefits of solar energy in different regions and contexts [25,26]. For example, Lama et al. estimated the job opportunities, the contribution of local entities, and the transfer of knowledge and skills with financial support from Chinese enterprises for solar energy in Sub-Saharan Africa [27]. García-Valladares and Ituna-Yudonago assessed the carbon footprint and economic co-benefits from solar energy for water heating systems in residential buildings in Mexico, Costa Rica, and the Democratic Republic of the Congo (DRC) [28]. Ren et al. looked at using the hybrid PV-battery system in residential units, showing that the households would effectively contribute to the self-consumption of on-site electricity generation or improve grid load control by providing additional grid electrical peak demand while improving the air quality, energy security, and load reduction [29]. In yet another study, Kim et al. revealed a significant reduction in energy consumption from using a hybrid renewable energy system (HRES) over existing gas-fired boilers or centralized heat pumps for a net-zero community in Jincheon, South Korea [30].

In recent years, the tools and types of co-benefits assessed in relevant research have grown increasingly diverse. For example, recent work has looked at the human well-being co-benefits from the implementation of the solar electrification projects for three electrification projects (grid extension, centralized hybrid, and solar home systems) in four remote communities in Malaysia, Cambodia, and Myanmar [31]. Taking a slightly different approach to co-benefits analysis, Lo et al. used Heilmann’s experimentation under a top-down hierarchy framework to identify synergies between solar energy and sustainable development, concluding that a qualitative assessment of the characteristics of co-benefits of solar energy policies could help local governments leverage solar energy for sustainable development in China [32]. In a study that further demonstrates the expanding scope of inquiry, Nutu et al. quantified the co-benefits of solar mini-grids to rural Ghanaian islands, including mediated impacts of heatwaves through the use of fans, reduced harmful gases from excessive burning of wood, and reduced social vices at night found that full recognition of an array of co-benefits of solar mini-grids offer more to sub-Saharan African rural populations than meeting development objectives alone [33]. 

### 1.2. Research Gaps and Originality Highlights

From the above discussion, it is clear that recognizing and quantifying co-benefits can spark interest in deploying solar energy projects. It is, nonetheless, less apparent how easily the tools and techniques used to quantify these benefits could be incorporated into decision-making processes and thereby achieve action on the ground. Part of the reason that research on co-benefits might have limited impacts on policy and action is that quantifying a full range of benefits is challenging. Those challenges, in turn, have led to the following gaps.

1. Existing co-benefits studies on a shift to a low-emission path through renewable energy mainly focus on costs. While costs are important, a failure to recognize benefits, particularly benefits that outweigh the costs (e.g., public health), can lead to flawed policy and project recommendations. Solar energy, especially in coal-dependent countries such as Mongolia, can bring multiple benefits such as improving local air quality, enhancing the local economy, and boosting energy security that can alter the decision-making calculus.
2. Although previous studies have highlighted some kinds of co-benefits, much of the research relies on tools and techniques that can be a black box for decision-makers. This is partially due to the inherent complexity of the modelling techniques. It is, however, also a function of a failure to develop tools that can be grasped with relative ease by decision-makers and their staff.

To fill the above research gaps, this study aims to place the emphasis on benefit estimation and open up the black box in ways that can shed light on how multiple co-benefits (such as improved air quality, health, and local economy) can be estimated. Especially, to fill the later gap, the study seeks to make co-benefits methods accessible with an easy-to-use spreadsheet-based simulation tool. The tool is employed to estimate the environmental, health, and economic co-benefits of using solar electricity and heat generation in the Ger area in UB, Mongolia. The assessment is based on calculating avoided emissions from a suggested project by the Financial UNEP Initiative to the local government, covering 100 MW solar electricity, including rooftop PV and community grids, and replacing heating load demand from existing HOBs for 20,000 households [5]. It is assumed that the amount of the generated power and heat from this project can meet the demand load requirement of the targeted society in this area. Toward this end, the spreadsheet tool simulates the hourly electricity and thermal energy generation from both solar panels and water heaters. The results are further processed to estimate the prevented disability-adjusted life years (DALYs) from the avoided emissions and associated economic benefits. The tool makes it possible to generate results that can inform policy. 

The remainder of this paper is organized as follows: the analytical methods are explained in Section 2. Section 3 covers the scenarios definitions, and Section 4 includes the results and discussion that follow from the conclusion.

## 2. Modeling Framework Development

To evaluate the potential of solar power and heat generation in UB and estimate the associated co-benefits from the utilization of solar energy in UB, the following steps were taken:

### 2.1. Environmental Benefits Assessment

**Figure 3:**
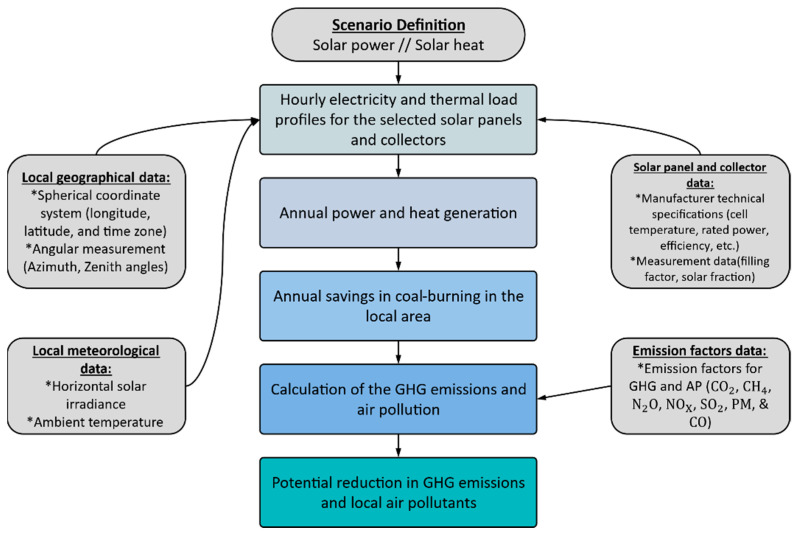
Calculation flow in the assessment of the environmental benefits (Created by the authors).

Figure 3 shows the calculation process for estimating the avoided emissions, including GHGs and other pollutants.

The potential of the avoided emissions after the utilization of solar energy can be estimated as follows:

(1) $$ER=ES_{E}\cdot EF_{E,\;coal}+ES_{H}\cdot EF_{H,\;coal}\;$$

(2) $$ES_{E}=\underset{t}{\sum }P_{PV,t}\cdot A_{Pv}$$

(3) $$ES_{EH}=\underset{t}{\sum }Q_{H,t}\cdot N$$

For the precise estimation of the hourly power ($P_{PV}$) and heat generation ($Q_{H}$), it is necessary to calculate the exact amount of solar radiation incident received by a tiled surface in a specific locale such as UB. The solar incident on the tiled surface of a solar panel or collector in the local area should be estimated by calculating its three main types of radiations, including beam, diffuse, and reflected from the ground. The detailed formulas used to calculate solar hourly power and heat in this study is represented in Table 1. $\bar{G_{T}}$ and $T_{C}$ are unknown parameters that need to be calculated further; the remaining parameters are manufactured data based on different PV arrays. After obtaining the $\bar{G_{T}}$, the solar radiation incident on the titled surface, the thermal heat generated by the solar collector can be calculated.

**Figure 4:**
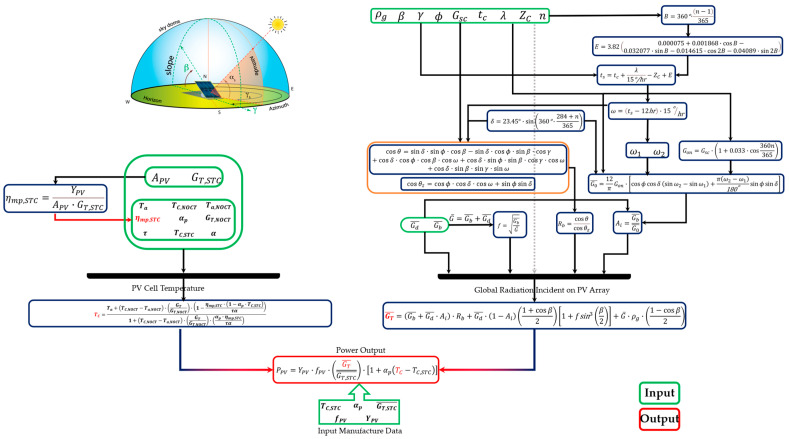
Calculation flow in solar power estimation (Created by the authors).

Figure 4 shows the calculation flow used in solar power estimation.

### 2.2. Public Health Benefits Assessment

Deaths, years of life lost (YLLs), years lived with disability (YLDs), and death average life years (DALYs) are all metrics used to evaluate the health burden of pollution. Deaths and YLLs are mortality metrics, while YLDs are used to assess morbidity, and DALYs are used to calculate overall mortality and morbidity. In this study, DALYs were selected as a metric to illustrate the mortality from different diseases. The effect estimates for DALYS were measured as the percent change in the health outcome per every unit change in the particulate matter (PM) concentrations. Despite the lack of data, the findings suggest that PM released at UB has substantial negative consequences on public health. Table 2 shows the most significant diseases in UB in terms of lost life years, based on data from the Global Burden of Disease Website. It is worth noting the magnitude of the impacts of Ischemic Heart Disease (IHD), Acute Lower Respiratory Infections (LRI), and stroke: these three endpoints account for approximately a third of all lost life years.

The morbidity and mortality are calculated as a function of relative risk. The population attributable risk fraction (*PAF*) for each disease is calculated using relative risk. In this case, *PAF* stands for the proportion of background disease caused by PM_2.5_ exposures, which is defined as [38]:

(4) $$PAF_{D,p}=\frac{RR_{D,p}-1}{RR_{D,p}}$$

The relative risk of the disease in question at the exposure level of interest is denoted by the *RR*. The *DALYs* attributed to the particular pollutant in a given year is calculated by multiplying each disease-specific *PAF* by the disease’s *DALYs*. The following equation can calculate the *DALYs* attributable to the selected disease (*D*) in the exposure group (*p*) in each scenario [39]:

(5) $$DALYs_{D,p}\left[\frac{y}{y}\right]=DALYs_{D}\left[\frac{y}{y}\right]\times PAF_{D,p}$$

The *DALYs* averted from the intervention scenario can be estimated using the following formula:

(6) $$ADALYs_{D,p,}\left[\frac{y}{y}\right]=DALYs_{D,p,B}-DALYs_{D,p,I}$$

where, $ADALYs_{D,p}$ represents the averted DALYs from the intervention scenario. 

In this study, PM is considered the main air pollutant that is contributing to the burden of diseases in UB. Dose-response curves for mortality risk of PM_2.5_ are derived from integrated exposure-response data provided by the Global Burden of Disease [37]. Linear regression was used to adapt a functional form of the collected data based on the concentration of PM_2.5_ ($\mu g/m^{3}$), as expressed by:

(7) $$RR=\alpha +\beta \left({\mathrm{PM}}_{2.5}\right)$$

The estimated values of α and β, using upper and lower uncertainty bounds at 95% confidence level, are given in Table 3.

### 2.3. Economic Benefits Assessment

The economic burden of disease has been assessed using through the different approaches, such as willingness to pay and the cost of illness [38]. In this study, the term “economic benefits” refers to the average *GDP* level that an individual in optimal health may achieve in a given year in a given country. Therefore, the monetized benefits of averted DALYs can be calculated using the following formula [39]:

(8) $$ExtraGDP_{D,p}\left[\frac{\$}{y}]=GDP[\$/y\right]\times ADALYs_{D,p}\left[\frac{y}{y}\right]$$

Since 1960, classical economists have debated the relationship between economic growth and unemployment. This relationship can be analyzed by using Okun’s law [40]. Okun’s law is a statistical relationship that draws upon regression to estimate the relationship between unemployment and economic growth. Depending on how fast the economy grew, the regression coefficients used to solve unemployment change may differ [41]. In this study, Okun’s law was employed to evaluate the statistical relationship between the unemployment rate and the financial benefit from the DALYs averted, which can be expressed as follows:

(9) $$\Delta u_{c,n}\left[\%\right]=\frac{\beta \times Saving_{D,p}/Pop}{GDP}\times 100$$

*β* is the Okun’s law coefficient estimated at 0.3705, using the historical data on the unemployment rate, *GDP*, and population in Mongolia, collected over the last 30 years. It is noted that the major reduction in the unemployment rate driven by the economic growth (*GDP*) is a result of ongoing increases in the size of the labor force and the level of productivity caused by the averted DALYs.

**Figure 5:**
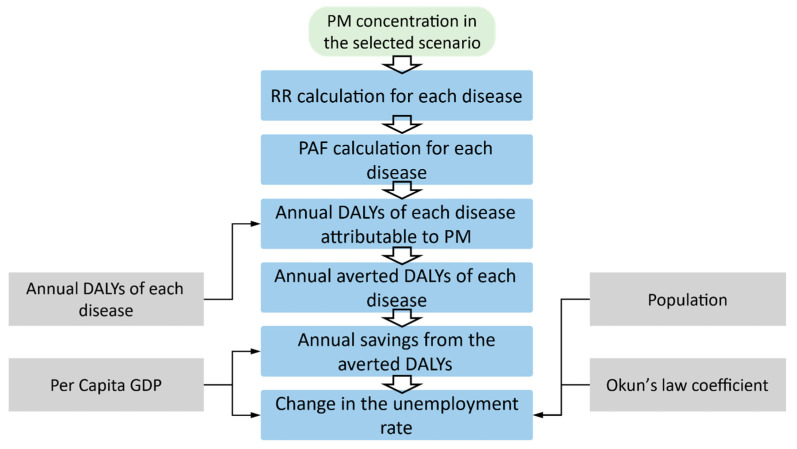
Calculation flow in health–economic benefits assessment (Created by the authors).

The overall calculation flow in this estimation is shown in Figure 5. As shown in this figure, the avoided PM concentration calculated in the environmental benefits assessment section is directly used to estimate the avoided health risk from the PM exposure, which is further used to quantify the averted DALYs. In addition, the macroeconomic and demographic data such as the per capita GDP, and population are used to estimate the change in the unemployment rate after implementing the solar project in UB.

The utilization of solar electricity and heat can also bolster both regional and national energy security in Mongolia. Saving in burning coal can reduce reliance on coal imports in the near future. To quantify the amount of burning coal, the following formula can be used:

(10) $$FS\left[t/h\right]=\frac{ES_{E}+ES_{H}}{LHV}$$

**Figure 6:**
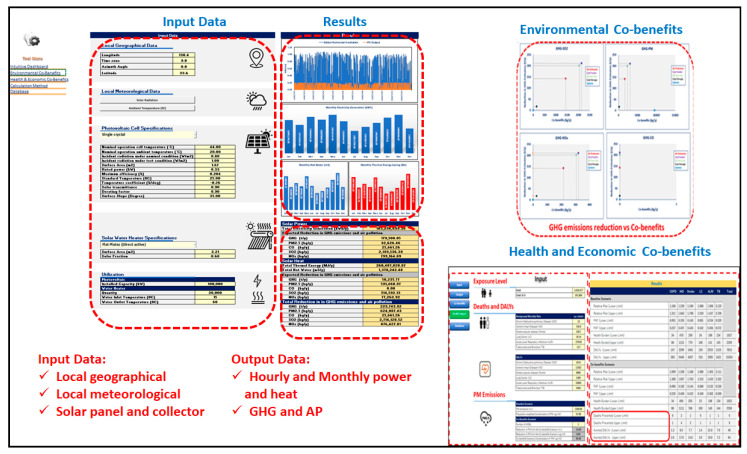
Overall view of the co-benefits assessment spreadsheet tool (Created by the authors).

The above formulas were then entered into the spreadsheet model to make it easy for users to quantify the multiple benefits (energy savings, environmental, local economy, and health) of implementing the solar energy (power and heat) project in UB. The main inputs include technical specifications of the solar panels, meteorological data, local geographical data, and emission factors. The tool also consists of an intuitive dashboard that allows for choosing two types (single and monocrystal) of solar panels and two types (flat plates and evacuated) of solar water heater collectors to estimate the hourly power and heat generation for an intervention scenario. The co-benefit assessment part of the tool provides an environmental–health–economic benefit based on the total amount of electricity and thermal energy generated from the proposed scenario, depicted in Figure 6.

## 3. Scenario Definition and Input Data

To evaluate the co-benefits from both solar power and heat in UB, the following intervention scenarios were considered [5]:

- Solar power scenario: 100 MW solar electricity, including both rooftop PV and community grids, can be installed in the near future to reduce the peak-load burden on the grid. The emission factors used in this scenario include those commonly used to calculate the amount of emissions in the power sector.
- Solar heat scenario: Solar thermal heaters will be provided to 20,000 households, replacing the existing HOBs. The emission factors used in this scenario include those used to calculate the amount of emissions from HOBs.

Table 4 shows the input data used to estimate the hourly power and heat generation from the different photovoltaic and collector types in the article. Although the tool can be used to evaluate several types of solar photovoltaics and collectors, the co-benefits analysis considers single-crystalline PV and flat plate collectors with the technical specification given in Table 4.

**Figure 7:**
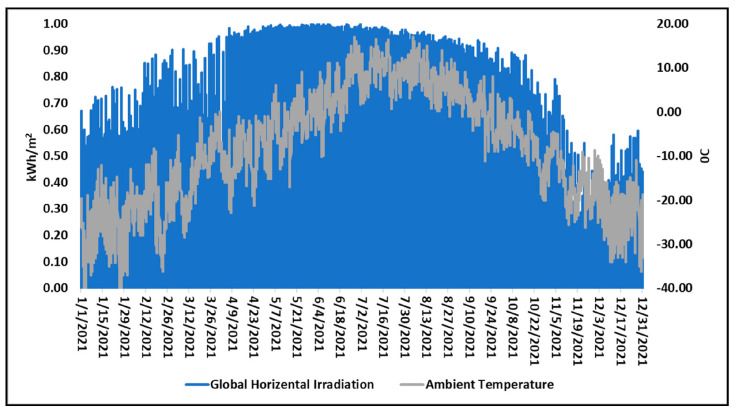
Hourly solar irradiation and ambient temperature in UB [44].

Figure 7 represents the collected hourly data on solar irradiation and ambient temperature in UB in 2021, using the Typical Meteorological Year (TMY) date from the European Energy Efficiency Platform (E3P) [44]. It can be observed from this figure that, despite freezing winters, UB has bountiful solar radiation, which peaks at more than 1 kWh/m^2^ during the warm season with more than an annual average of 2800 h of sunshine.

The emission factors used in the calculation are given in Table 5.

## 4. Results and Discussion

**Figure 8:**
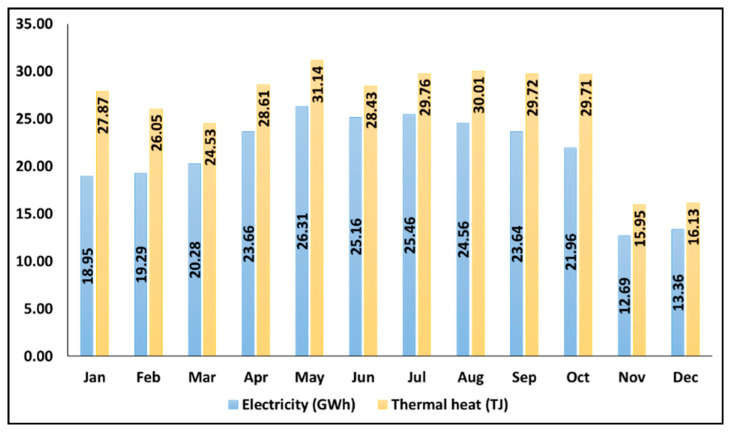
Monthly average electricity and thermal heat generation from the inversion scenarios in UB (Estimated by the authors).

Figure 8 shows the monthly average electricity and thermal generation from the intervention scenarios in UB. The maximum amount of both solar electricity and heat can be achieved in May, which are around 23.66 (GWh) and 31.14 (TJ), respectively. The annual electricity and thermal energy are estimated at 255.3 (GWh/y) and 317.9 (TJ/y), respectively.

Table 6 shows the annual electricity and heat generation and also expected reduction in GHG emissions and other air pollutants for each scenario.

**Figure 9:**
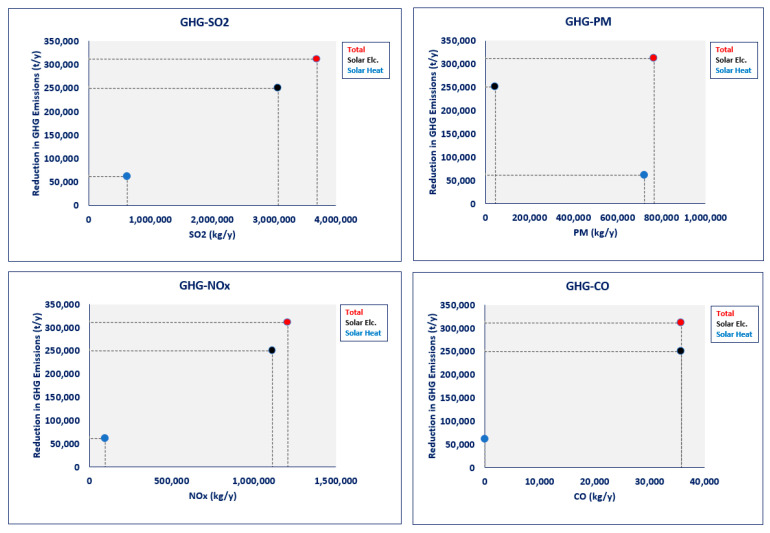
GHG emissions reduction vs. environmental co-benefits (Estimated by the authors).

The estimated reductions in GHG emissions and air pollutants in the solar power scenario are higher than in the solar heat scenario. Figure 9 illustrates the variation of the GHG emission reduction and other pollutants associated with the above intervention scenarios. It can be observed from this figure, although the solar heat scenario represents a significant reduction in PM_2.5_, the expected co-benefits from the solar power scenario for SO_2_, NOx, and CO would be much higher than this scenario.

To assess the current situation in the baseline scenario, PM data for UB were obtained from the Air Quality Analysis of Ulaanbaatar [7]. The article assumes the population-weighted annual average exposure of PM in UB is estimated at 57 ug/m3 with a yearly emission of 1300 tons [46]. Table 6 shows the annual DALYs’ estimated values attributable to PM emissions in the baseline scenario. According to the model results, about 767 t/y reduction in PM emissions would be expected in UB from the combined power and heat scenario. This amount corresponds to an approximately 27.36 ug/m3 decrease in the population-weighted concentration of PM in UB. The health co-benefits from deploying the intervention scenario are reported in Table 7.

**Figure 10:**
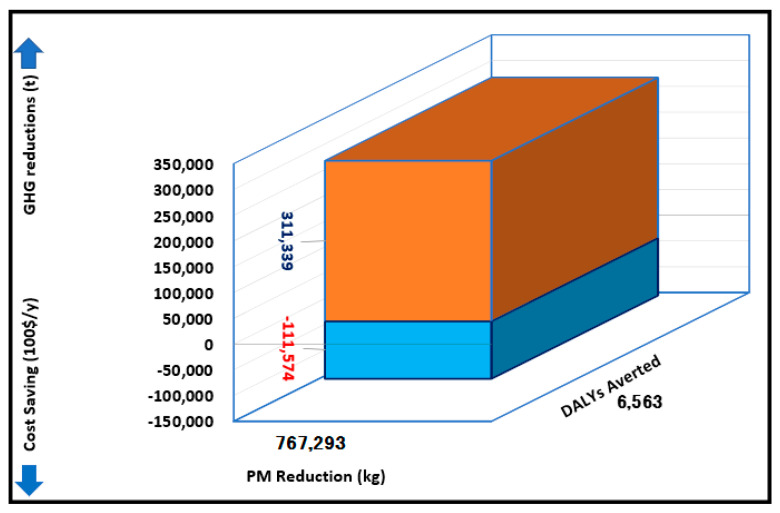
Combined co-benefits from the intervention of the combined scenario (Estimated by the authors).

Figure 10 represents the combined co-benefits benefits from the intervention scenarios in UB. It can be observed from this figure that installing 100 MW of solar power and 20,000 solar water heaters in UB results in reducing about 311,000 tons of GHGs and about 767 tons of PM; this translates to nearly 4545 DALYs and an annual saving of 11.2 million USD for UB’s economy.

The total expected co-benefits from the combined scenario are given in Table 8.

**Figure 11:**
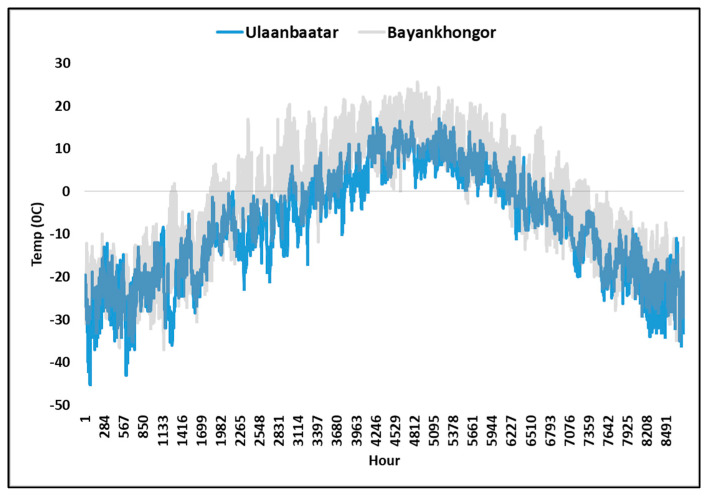
Hourly ambient temperature in UB and Bayankhongor.

In order to assess the impact of the local meteorological factors, such as the ambient temperature on the performance of the solar photovoltaic and water heater collectors, the expected co-benefits from the implementation of the suggested scenarios in UB were com-pared with another city in Mongolia, Bayankhongor, which has moderated ambient temperature. The result of the comparison between the ambient temperature and expected co-benefits are represented in Figure 11 and Table 9, respectively. It can be observed that, the ambient condition significantly influences the averted DALYs in this region.

## 5. Policy Implications

The results of the modeling could help to strengthen future climate change and air pollution policies in UB and other parts of Mongolia. This potential is particularly great because climate change and air pollution are closely interlinked. Those interlinkages open opportunities to strengthen synergies between climate and air pollution policy. As noted earlier in the article, Mongolia’s policymakers have made some efforts along these lines. For example, in November 2019, Mongolia pledged to reduce GHGs by 22.7 percent by 2030 in its updated Nationally Determined Contributions (NDC). With that updated NDC, Mongolia committed to implement policies and measures in the energy and five other sectors with potential co-benefits. In addition, air pollution policies with similar promises have been promulgated in UB. 

Another opening for co-benefits is the national program for the reduction of air and environmental pollution that is scheduled to reduce pollution by 80% compared to 2016 over two phases. Last but not least, some of the recommendations being considered in Mongolia for the short- and long-term include carbon pricing and the long-term banning of raw coal in favor of renewable energy sources that could also deliver co-benefits. While the above commitments and actions are promising, there is nonetheless scope to do more. For example, solar energy is a zero-emission energy source that has been recognized as a key building block for climate-neutral energy infrastructure. However, solar energy’s contributions to air quality have not been explicitly highlighted in relevant decision-making processes. Making findings such as the significant reductions in PM from renewable energy would arguably strengthen both climate and air policy in Mongolia. They could also contribute to a further strengthening of Mongolia’s NDCs and efforts to transition onto net-zero development paths.

In addition, efforts will need to focus on building bridges across the agencies and divisions that are responsible for climate change, air pollution, and health. Too often, siloed institutions and decision-making processes leave co-benefits underappreciated and unrealized. One way to make them more visible in decisions is to equip decision-makers working on climate change, air pollution, and health with accessible tools. This article, therefore, also takes an important step in that direction.

## 6. Conclusions

This study contributed to existing research by demonstrating a quantitative analytical modeling approach to comprehensively quantify the multiple environmental, health, and economic benefits from the installation of 100 MW solar electricity and providing solar thermal heaters to 20,000 households in this region. The results revealed a significant reduction in GHG emissions and air pollution, which can help to prevent nearly 6500 disability-adjusted life years (DALYs) and provide an annual saving of USD 7.7 million for the local economy. These kinds of estimates may persuade policymakers to take action on climate change and air quality.

While the study has considerable potential to motivate action, it also opens opportunities for further inquiry that are not a focal point in the article. Potentially fruitful areas for further research include a wider variety of scenarios that could reflect on issues such as size optimization for solar power and heat systems or a hybrid (wind–solar–battery) renewable energy system in the Ger area. An additional field of study that could be linked more explicitly to the co-benefits analysis includes an economic analysis of costs or cost-benefit analysis of different interventions. Providing benefit-cost ratios may prove even more persuasive to policymakers.

## Figures and Tables

**Table 1:** Detailed calculation steps of solar power and heat generation [34,35,36].

Solar Power Generation
$P_{PV}=Y_{PV}\cdot f_{PV}\cdot \left(\frac{\bar{G_{T}}}{\bar{G_{T,STC}}}\right)\cdot \left[1+\alpha_{p}\left(T_{C}-T_{C,STC}\right)\right]$ $T_{C}=\frac{T_{a}\;+\;\left(T_{C,NOCT}-T_{a,NOCT}\right)\cdot \left(\frac{G_{T}}{G_{T,NOCT}}\right)\cdot \left(1-\frac{\eta_{mp,STC}\cdot \left(1-\alpha_{p}\cdot T_{C,STC}\right)}{\tau \alpha }\right)}{1+\left(T_{C,NOCT}-T_{a,NOCT}\right)\cdot \left(\frac{G_{T}}{G_{T,NOCT}}\right)\cdot \left(\frac{\alpha_{p}\cdot \eta_{mp,STC}}{\tau \alpha }\right)}$ $G_{T}=\left(\bar{G_{b}}+\bar{G_{d}}\cdot A_{i}\right)\cdot R_{b}+\bar{G_{d}}\cdot \left(1-A_{i}\right)\left(\frac{1+\cos\beta }{2}\right)\left[1+f\sin^{3}\left(\frac{\beta }{2}\right)\right]+\bar{G}\cdot \rho_{g}\cdot \left(\frac{1-\cos\beta }{2}\right)$ $A_{i}=\frac{\bar{G_{b}}}{\bar{G_{0}}}$ $\bar{G_{0}}=\frac{12}{\pi }G_{on}\cdot \left[\cos\phi \cos\delta \left(\sin\omega_{2}-\sin\omega_{1}\right)+\frac{\pi \left(\omega_{2}-\omega_{1}\right)}{180{}^{\circ}}\sin\phi \sin\delta \right]$ $G_{on}=G_{sc}\cdot \left(1+0.033\cdot \cos\frac{360n}{365}\right)$ $R_{b}=\frac{\cos\theta }{\cos\theta_{z}}$ $f=\sqrt{\frac{\bar{G_{b}}}{\bar{G}}}$ $\bar{G}=\bar{G_{b}}+\bar{G_{d}}$ $\delta =23.45{}^{\circ}\cdot \sin\left(360{}^{\circ}\cdot \frac{284+n}{365}\right)$ $\omega =\left(t_{s}-12\;h\right)\cdot 15{}^{\circ}/h$ $t_{s}=t_{c}+\frac{\lambda }{15{}^{\circ}/h}-Z_{C}+E$ E=3.82(0.000075+0.001868·cosB−0.032077·sinB−0.014615·cos2B−0.04089·sin2B) $B=360{}^{\circ}\cdot \frac{\left(n-1\right)}{365}$ $\begin{array}{l} \cos\theta =\sin\delta \cdot \sin\phi \cdot \cos\beta -\sin\delta \cdot \cos\phi \cdot \sin\beta \cdot \cos\gamma +\cos\delta \cdot \cos\phi \cdot \cos\beta \cdot \cos\omega +\cos\delta \cdot \sin\phi \cdot \sin\beta \cdot \cos\gamma \cdot \cos\omega \\ +\cos\delta \cdot \sin\beta \cdot \sin\gamma \cdot \sin\omega \end{array}$
Solar heat generation
$Q_{H}=\bar{G_{T}}\cdot \delta_{fn}$ $m_{w}\cdot C_{p,w}\cdot \left(T_{e}-T_{i}\right)=A_{c}\cdot Q_{H}$

**Table 2:** Total DALYs of the most critical diseases in UB, Mongolia [37].

	DALYs
Chronic Obstructive Pulmonary Disease (COPD)	383
Ischemic Heart Disease (IHD)	5448
Cerebrovascular Disease (Stroke)	4207
Lung Cancer (LC)	510
Acute Lower Respiratory Infections (ALRI)	3285
Tuberculosis and Bronchus (TB)	1421

**Table 3:** Estimated RR linear equation (95% CI) (Estimated by the authors).

	α	β	R^2^
IHD			
Low	0.0025	1.0767	0.884
UP	0.0108	1.1095	0.9141
Stroke			
Low	0.0019	1.0541	0.983
Up	0.0104	1.0489	0.8886
COPD			
Low	0.0016	1.0084	0.9999
Up	0.0055	1.0691	0.9837
LC			
Low	0.0018	0.9849	1.000
Up	0.0075	1.0746	0.9962
ALRI (Ave.)	0.0108	0.9249	0.9957
TB (Ave.)	0.0225	0.8426	0.9922

**Table 4:** Technical specification of the photovoltaic and solar collector used in this research.

**Solar Photovoltaic** [42]	
Nominal operation cell temperature (°C)	44.00
Incident radiation under nominal condition (W/m^2^)	0.80
Surface area (m^2^)	1.67
Rated power (kW)	0.33
Temperature coefficient (%/deg)	−0.26
Solar transmittance	0.90
Derating factor	0.901
Solar Collector [43]	
Surface area (m^2^)	1.98
Solar fraction	0.74

**Table 5:** Emission factors used in this study [45].

Emission Factors	Electricity (g/kWh)	Thermal (kg/mmBtu)
CO_2_	971.51	93.28
N_2_O	0.02	0.001
CH_4_	0.11	0.01
PM_2.5_	0.18	2.40
CO	0.14	0.00
SO_2_	12.00	2.08

**Table 6:** Power/heat and expected reductions in GHG emissions and AP in each scenario (Estimated by the authors).

*Solar Power Scenario*	
Total Electricity Generation (GWh/y)	255.3
Expected reduction in GHG emissions and air pollution	
GHG (1000 t/y)	250.0
PM_2.5_ (t/y)	45.4
CO (t/y)	35.7
SO_2_ (t/y)	3063.6
NOx (t/y)	1113.1
*Solar Heat Scenario*	
Total Thermal Energy (TJ/y)	317.9
Expected reduction in GHG emissions and air pollution	
GHG (1000 t/y)	61.3
PM_2.5_ (t/y)	721.8
SO_2_ (t/y)	628.0
NOx (t/y)	94.3
*Total Reduction in GHG emissions and air pollution*	
GHG (1000 t/y)	311.3
PM_2.5_ (t/y)	767.2
CO (t/y)	35.7
SO_2_ (t/y)	3691.6
NOx (t/y)	1207.5

**Table 7:** DALYs of different diseases caused by PM emissions before and after the intervention scenarios.

	COPD	IHD	Stroke	LC	ALRI	TB	Total
Before							
Relative Risk (Lower Limit)	1.100	1.239	1.169	1.088	1.306	2.125	
Relative Risk (Upper Limit)	1.311	1.842	1.768	1.520	1.437	2.338	
PAF (Lower Limit)	0.091	0.193	0.145	0.081	0.234	0.529	
PAF (Upper Limit)	0.237	0.457	0.434	0.342	0.304	0.572	
DALYs (Lower Limit)	147	2299	1401	120	2533	1315	7815
DALYs (Upper Limit)	383	5448	4207	510	3285	1421	15,254
After							
Relative Risk (Lower Limit)	1.046	1.154	1.099	1.027	1.118	1.368	
Relative Risk (Upper Limit)	1.157	1.351	1.299	1.251	1.230	1.505	
PAF (Lower Limit)	0.044	0.133	0.090	0.026	0.105	0.269	
PAF (Upper Limit)	0.136	0.260	0.230	0.200	0.187	0.336	
DALYs (Lower Limit)	71	1589	874	39	1138	668	4380
DALYs Upper Limit)	219	3094	2228	299	2017	833	8690
Averted DALYs (Lower Limit)	76	711	526	81	1395	647	3435
Averted DALYs Upper Limit)	163	2354	1979	211	1268	588	6563

**Table 8:** Expected environmental–health–economic benefits from the combined scenario (Estimated by the authors).

Co-Benefits	Combined Scenario
Reduction in GHG emissions (1000 t)	311.4
Reduction in PM emission (t)	767.2
Averted DALYs	6563
Avoided health cost (M$)	11.2
Reduction in the unemployment rate (%)	0.136
Energy security in terms of fuel-saving (t/y)	117

**Table 9:** Comparison of the expected co-benefits from the combined scenario in UB and Bayankhongor (Estimated by the authors).

Co-Benefits	Ulaanbaatar	Bayankhongor
Total Electricity Generation (GWh/y)	255.3	275.2
Total Thermal Energy (TJ/y)	317.9	336.4
Reduction in GHG emissions (1000 t)	311.4	314.7
Reduction in PM emission (t)	767.2	809.1
Averted DALYs	6563	7268

## Data Availability

Not applicable.

## Nomenclature

ER	Total annual reduction in emissions (Mt)
$ES_{E}$	Annual power generation from the solar panel (kWh/y)
$ES_{EH}$	Annual heat generation from the solar panel (GJ/y)
$EF_{Elc,\;coal}$	Pollutants emission factors of coal per unit of electricity generated from the grid (g/kWh)
$EF_{H,\;coal}$	Pollutants emission factors of coal per unit of thermal energy (g/GJ)
$P_{PV}$	Hourly electricity generation from each square meter of the solar panel (kWh/m^2^)
$A_{Pv}$	Total surface area for solar power generation (m^2^)
$Q_{H,t}$	Hourly thermal energy generation from each solar collector (MJ/h)
N	Quantity of the solar water heaters
*t*	Time period (h)
$Y_{PV}$	the rated capacity of the PV array; namely, the power output under standard test conditions (luminous intensity: 1000 $W/m^{2}$; temperature: 25 °C; air mass: 1.5) [kW]
$f_{PV}$	the PV derating factor (%)
$\bar{G_{T}}$	the solar radiation incident on the PV array in the current time step [$\mathrm{kW}/m^{2}$]
$\bar{G_{T,STC}}$	the incident radiation at standard test conditions [1 $\mathrm{kW}/m^{2}$]
$\alpha_{p}$	the temperature coefficient of power [%/°C]
$T_{C}$	the PV cell temperature in the current time step [°C]
$T_{C,STC}$	the PV cell temperature under standard test conditions [25 °C]
$\bar{G_{b}}$	the beam radiation [$\mathrm{kW}/m^{2}$]
$\bar{G_{d}}$	the diffuse radiation [$\mathrm{kW}/m^{2}$]
$\omega_{1}$	hour angle at the beginning of the time step [°]
$\omega_{2}$	hour angle at the end of the time step [°]
n	the day of the year [a number between 1 and 365]
$G_{sc}$	the solar constant [1.367 $\mathrm{kW}/m^{2}$]
$\rho_{g}$	the ground reflectance, which is also called the albedo [%]
$t_{s}$	the solar time [h]
$t_{c}$	civil time in hours corresponding to the midpoint of the time step [h]
λ	longitude of the location [°]
$Z_{C}$	the time zone in hours east of GMT [h]
E	the equation of time [h], the calculation method of it is as below:
θ	the angle of incidence [°]
β	the slope of the surface [°]
γ	the azimuth of the surface [°]
ϕ	the latitude [°]
δ	the solar declination [°]
ω	the hour angle [°]
$T_{a}$	the ambient temperature [K]
$T_{C,NOCT}$	the nominal operating cell temperature [317 K]
$T_{a,NOCT}$	the ambient temperature at which the NOCT is defined [293 K]
$G_{T}$	the solar radiation striking the PV array [$\mathrm{kW}/m^{2}$]
$G_{T,NOCT}$	the solar radiation at which the NOCT is defined [0.8 $\mathrm{kW}/m^{2}$]
$\eta_{mp,STC}$	the efficiency of the PV array at its maximum power point under standard conditions [%] ($\eta_{mp,STC}=\frac{Y_{PV}}{A_{PV}\cdot G_{T,STC}}$, where $A_{PV}$ is the surface of the PV array [$m^{2}$], and $G_{T,STC}$ is equal to 1$\;\mathrm{kW}/m^{2}$)
$\alpha_{p}$	the temperature coefficient of power [%/°C]
$T_{C,STC}$	the cell temperature under standard test conditions [298 K]
τ	the solar transmittance of any cover over the PV array [%]
α	the solar absorptance of the PV array [%]
$\delta_{fn}$	the design solar fraction [30–60%]
$C_{p,w}$	the specific heat capacity of water [kJ/(kg °C)]
$T_{e}$	the required temperature of hot water [°C]
$T_{i}$	the initial temperature of inlet water [°C]
$A_{c}$	the area of heat collector [$m^{2}$]
*LHV*	the low calorific heating value of coal (Bitaminus in this research) [GJ/t]
$PAF_{D,p}$	the population attributable risk fraction of the disease (d) in the exposure group (*p*)
$RR_{D,p}$	the relative risk of
$DALYs_{D,p}$	DALYs attributable to the selected disease (D) in the exposure group (*p*)
$ADALYs_{D,p}$	Averted DALYs from the intervention scenario for the disease (D) in the exposure group (*p*)
*B*	Baseline scenario
*I*	Intervention scenario
GDP	the per capita GDP [$]
$Saving_{D,p}$	Monetized benefits of the DALYs averted of the disease (d) in the exposure group (*p*)
$\Delta u_{c,n}$	Change in the unemployment rate by introducing solar energy projects
β	Okun’s Law coefficient, which is estimated by using historical data on unemployment rates and GDP
*Pop*	Population
ExtraGDP	Average GDP level that an individual in optimal health may achieve in a given year in a given country [$]
$\;ES_{E}$	Annual electricity generated from the solar photovoltaic [GJ]
$ES_{H}$	Annual thermal energy generated from the solar collector [GJ]
FS	Fuel saving [t]
